# Reversing the Negative Genomic Effects of Aging with Short-Term Calorie Restriction

**DOI:** 10.1100/tsw.2001.256

**Published:** 2001-10-12

**Authors:** Stephen R. Spindler

**Affiliations:** Department of Biochemistry, University of California, Riverside, Riverside, CA 92521, USA

**Keywords:** calorie restriction, microarray, gene expression, aging, age-related diseases, cancer, drug discovery

## Abstract

According to government figures, total health care spending in the U.S. in 1999 was $1.316 trillion. The government projects an increase in health care costs to $2.176 trillion by 2008. If we project this growth rate to 2020, health care costs will reach $4.009 trillion. Today, people often spend more health care dollars during the last year of their lives than in all previous years combined. Medical treatment in the last few years of life is usually very expensive and often futile. With the baby-boom generation now moving through middle age, the prescription for the U.S. health care system will be disastrous unless we learn how to keep people healthier longer. This dramatic increase in health care costs leaves us with only one acceptable alternative to rationed health care or financial ruin — to discover interventions that make people functionally younger, healthier, and less susceptible to debilitating, age-related diseases.

Scientists have known since the 1930s that a relatively simple dietary treatment termed "caloric restriction" was capable of reducing the incidence and severity of age-associated diseases such as cancer and heart disease and, at the same time, actually extending the maximum life span of mammals. A number of studies suggests that these diets may also be effective for humans.

On September 11$^{th}$, a paper was published in the *Proceedings of the National Academy of Sciences* (PNAS) from my laboratory[1]. My research group has been probing the mechanism for the almost magical, life-extending effects of calorie restriction using advanced gene chip technology. The results of this study indicate for the first time that calorie restriction started late in life, in very old animals, rapidly reverses many of the negative changes in gene expression that had accumulated over a lifetime of normal eating. These results mean that it is possible to test pharmaceuticals and other compounds for the anti-disease, anti-aging effects of calorie restriction in a matter of weeks instead of years. These findings are already drastically accelerating the search for anti-aging treatments that will reduce the incidence and severity of age-related diseases. In addition to my duties as Professor in the Department of Biochemistry at the University of California at Riverside, and I am also Chief Scientific Officer for LifeSpan Genetics Inc., which is attempting to take our discoveries from the laboratory to widespread use.

The ability of caloric restriction to retard aging and extend maximum life span in mammals was first discovered by Cornell University nutritionist Clive M. McCay. McCay’s longest-lived, calorie-restricted rat lived for 48 months, while his longest-lived, control-fed animal died at the age of only 30 months[4,5]. In later experiments at the Institute For Cancer Research in Philadelphia, Morris Ross was able to keep calorie-restricted rats alive for more than 59 months, about 180 years in human terms[6]! Over the years, dozens of scientists have demonstrated that caloric restriction extends maximum life span in every species studied to date[9]. Ongoing studies, the most extensive taking place at the National Institutes of Health, indicate that this method retards aging and associated diseases in primates, which differ from humans by only 2% at the DNA level[7]. These and other observations suggest that calorie restriction will slow aging and improve the health of humans.

There is evidence of a beneficial role for reduced caloric intake on the incidence of prostate cancer, colon cancer, and Parkinson’s and Alzheimer’s diseases in humans. Occurrence of these diseases appears to be lower in individuals who have lower caloric intakes. The eight men and women who lived for 2 years in the Biosphere 2 habitat consumed a diet that was nutritious but was reduced in calories due to unexpectedly low food production. Careful studies indicated that they were significantly more healthy during this period of caloric restriction[8,10,11]. Now, for the first time we have evidence from our studies that low calorie diets, initiated even very late in life, can actually reverse the majority of the negative effects on gene expression accumulating from a lifetime of overeating. This may mean that older people may be able to benefit rapidly from switching to a calorie-restricted diet. This idea is consistent with information that has been known to doctors and scientists for years. For instance, the health of overweight individuals and type II diabetics improves very rapidly when they begin low calorie diets, even before the fat mass is depleted[2,3]. New work from this laboratory shows, for the first time, that these improvements extend to the majority of the gene expression effects produced by caloric restriction.

Most scientists agree that genes play a major role in the onset and progression of the diseases of aging and of aging itself. Previously, most scientists had assumed that caloric restriction prevents deleterious age-related changes in gene expression from taking place. We examined aging changes in the expression of 11,000 genes in liver, and the modification of these changes by calorie restriction[1]. We began the studies with two groups of young mice and two groups of old mice. Some of the young and old mice ate almost as much food as they wanted their whole lives, although they were not given enough to get fat. Other groups of young and old mice were fed 50% less than they would like to eat throughout their lives, after weaning. Finally, we took a group of very old female mice (equivalent to 70- to 80-year-old humans) who had eaten nearly all they wanted their entire lives, and we said to them “okay ladies, the party's over, it's time to diet”. We underfed them a little for 2 weeks, and then underfed them to the same degree as the long-term calorie-restricted mice. When we compared the gene expression profiles in the livers of these mice, we found that aging produced changes in gene expression associated with increased inflammation, cellular stress and fibrosis, and reduced capacity for apoptosis, xenobiotic metabolism, normal cell division, and DNA replication. Long-term calorie restriction and, very surprisingly, the mere 4 weeks of short-term calorie restriction described above reversed the majority of these changes. Long- and short-term caloric restriction rapidly produced a gene expression profile consistent with decreased inflammation and cellular stress, increased apoptosis, improved metabolism of foreign chemicals, and better cardiovascular health. Thus, short-term caloric restriction actually rapidly reversed most of the negative consequences of a lifetime of overeating, and reproduced the benefits of a lifetime of undereating.

It is probably not practical to expect that many people will be able to undereat sufficiently to reap the rewards of caloric restriction. A more practical alternative is needed. Many people have recognized the importance of developing *calorie restriction mimetics*, or drugs that mimic the effects of calorie restriction. Our work indicates that gene chip analysis, used on animals and humans treated briefly with drugs or nutraceuticals, would be the fastest way of doing a first screening for compounds that mimic the effects of caloric restriction. LifeSpan Genetics is doing this kind of screening now. Thus, finding compounds capable of delaying the onset of age-related diseases, and even capable of extending our maximum life span are real possibilities for the first time.
